# A follow-up study of heroin addicts (VEdeTTE2): study design and protocol

**DOI:** 10.1186/1747-597X-2-9

**Published:** 2007-03-15

**Authors:** Federica D Vigna-Taglianti, Federica Mathis, Roberto Diecidue, Paola Burroni, Antonio Iannaccone, Fabio Lampis, Piergiorgio Zuccaro, Roberta Pacifici, Elisabetta Versino, Marina Davoli, Fabrizio Faggiano

**Affiliations:** 1 Piedmont Centre for Drug Addiction Epidemiology, ASL 5 – Grugliasco, Italy; 2 Department of Clinical and Experimental Medicine, Avogadro University, Novara, Italy; 3 Drug Abuse Treatment Centre, ASL TO 1, Turin, Italy; 4 National Institute of Health, Rome, Italy; 5 Department of Public Health, University of Turin, Italy; 6 Department of Epidemiology, ASL Rome E, Rome, Italy

## Abstract

**Background:**

In Italy, a large cohort study (VEdeTTE1) was conducted between 1998–2001 to evaluate the effectiveness of treatments in reducing mortality and increasing treatment retention among heroin addicts. The follow-up of this cohort (VEdeTTE2) was designed to evaluate the effectiveness of treatments on long-term outcomes, such as rehabilitation and social re-integration. The purpose of this paper is to describe the protocol of the VEdeTTE2 study, and to present the results of the pilot study carried out to assess the feasibility of the study and to improve study procedures.

**Methods:**

The source population for the VEdeTTE2 study was the VEdeTTE1 cohort, from which a sample of 2,200 patients, traced two or more years after enrolment in the cohort, were asked to participate. An interview investigates drug use; overdose; family and social re-integration. Illegal activity are investigated separately in a questionnaire completed by the patient. Patients are also asked to provide a hair sample to test for heroin and cocaine use. Information on treatments and HIV, HBV and HCV morbidity are obtained from clinical records. A pilot phase was planned and carried out on 60 patients.

**Results:**

The results of the pilot phase pointed out the validity of the procedures designed to limit attrition: the number of traced subjects was satisfactory (88%). Moreover, the pilot phase was very useful in identifying possible causes of delays and attrition, and flaws in the instruments. Improvements to the procedures and the instruments were subsequently implemented. Sensitivity of the biological test was quite good for heroin (78%) but lower for cocaine (42.3%), highlighting the need to obtain a hair sample from all patients.

**Conclusion:**

In drug addiction research, studies investigating health status and social re-integration of subjects at long-term follow-up are lacking. The VEdeTTE2 study aims to investigate these outcomes at long-term follow-up. Results of the pilot phase underline the importance of the pilot phase when planning a follow-up study.

## 1 Background

Apart from excess mortality [1-5], the health effects of heroin use are related to: the direct effects of drug use (e.g. overdose); concurrent psychiatric pathologies (e.g. suicide); injections (e.g. HIV, hepatitis, site infections); and the social effects (e.g. unemployment, criminal activity, prostitution, deterioration of family and social relations) [6].

In the short-term, drug abuse treatment aims to protect subjects from overdose and infections, and lower the risk of death. In the mid- to long-term, treatment is aimed at social re-integration and, therefore, rehabilitation. Cohort studies investigating the effects of treatment for heroin addiction (DARP, TOPS, NTORS, DATOS, Amsterdam Cohort Study) have been conducted in various countries. Data on heroin and cocaine use, overdose, mortality, crime and illegal activity have been evaluated in short-term follow-up studies [7-18]. Mortality data and substance use have been evaluated also in long-term follow-up [1,2,10,15,17,19-22]. However, few studies have assessed social rehabilitation and re-integration in mid- to long-term follow-up [15,19,23-25], and very few have evaluated the relationship between outcomes and drug abuse treatment [15,19].

In Italy, the first phase of a large multicentre cohort study on heroin addicts, known as VEdeTTE (Italian acronym for "Evaluation of Effectiveness of Treatments for Heroin Dependence") [26], was conducted between 1998 and 2001. The main aim of the study was to evaluate the effectiveness of treatments in reducing mortality and increasing retention in treatment. The first phase, referred to as VEdeTTE1, involved 115 drug abuse treatment centres of the National Health Service (NHS). The enrolled population consisted in 11,903 Italian heroin addicts, aged 18 years or older, who received treatment at the participating centres during the study period [26].

The follow-up of a sample of the VEdeTTE1 cohort was organized to evaluate the effectiveness of treatments with regard to long-term legal and illegal drugs use; overdose; family and social re-integration; and HIV, HBV and HCV morbidity. Forty-two NHS drug abuse treatment centres participated in this second phase, referred to as VEdeTTE2, which came to an end in December 2006. The assessment of long-term outcomes other than mortality involves tracing patients and obtaining their consent again, which requires good study design and well-organized field work. Moreover, in conducting a follow-up study several critical methodological issues must be considered, such as attrition and the accuracy of self-reported drug use.

To evaluate the feasibility of the VEdeTTE2 study and to ensure the quality of follow-up procedures, a pilot study was conducted in 2001. The purpose of this paper is to describe the VEdeTTE2 study design and protocol, and to present the results of the pilot study and the improvements to the protocol implemented in consequence of the pilot study.

## 2 VEdeTTE2 study protocol

### 2.1 Study population

The study population of the VEdeTTE2 study consist of a sample of patients enrolled in the VEdeTTE1 cohort between September 1998 and March 2001: all new patients and a random sample of re-entry and prevalent patients. The follow-up period is a minimum of two years between the first interview in VEdeTTE1 and the VEdeTTE2 follow-up interview. A sample size of 824 is required to find a statistically significant Relative Risk of 1.5, with an alpha error of 0.05, a beta error of 10% and an estimated prevalence of heroin users of 20%. However, based on available funds, it was decided to follow-up 2,200 patients.

### 2.2 Consent form and confidentiality of data

All procedures and instruments were approved by the Ethical Committee. A telephone number to contact the Ethical Committee is specified on the written consent form, a copy of which is given to the patient at the time of enrolment. Precautions are taken to guarantee data confidentiality. Data are identified using the same anonymous code assigned to the patient upon enrolment in VEdeTTE1. The possibility of matching the anonymous code with patient identity is restricted to the NHS treatment centres and the Regional Coordinating Centres. Moreover, patients are assigned to interviewers whom they have never met before, and they have the choice of being interviewed outside the NHS treatment centre. Finally, only aggregate results will be communicated to NHS treatment centres.

### 2.3 Tracing procedures

To minimize attrition and achieve a good contact rate, detailed procedures were devised. First of all, a reward is given to participating patients, as suggested in the international literature [27,28]. Patients are given 15 euro for the interview and 37 euro for the biological sample. Secondly, the interviewers are either external interviewers or NHS treatment centre personnel; they are trained in a one-day training session, and paid 129 euro for each interview and 15 euro for each refusal or drop-out. Thirdly, accurate tracing procedures were implemented. For patients still attending NHS treatment centres, the interviewer is introduced to the patient by a clinician at the treatment centre. For patients no longer attending the drug treatment centre, the interviewer contacts the patient with the help of treatment centre personnel. In the case of failure, life status and home address are obtained at the General Registry Office and, whenever possible, a phone number from the patient's acquaintances. Finally, a maximum of three attempts are made to contact the patient by letter sent to the last known address.

### 2.4 Interview and questionnaire

Participants are required to complete a questionnaire administered by an interviewer. The questionnaire consists of eleven sections: 1) civil status, educational level, accommodation, job (to be completed for refusals and drop-outs only); 2) HIV, HBV and HCV test results, other pathologies recorded in the case history; 3) changes in civil status, educational level and life events since the VEdeTTE1 interview (e.g. marriage, divorce, births); 4) self-reported pathologies; 5) illegal activity; 6) drug use since the VEdeTTE1 interview (frequency, dose, route of administration); 7) overdose; 8) use of legal drugs without a medical prescription; 9) drug use in the last 30 days (frequency, dose, route of administration); 10) risky behaviours; 11) educational level, accommodation, job, friendships, financial help, hobbies. The first section for refusals and drop-outs is completed from information in the clinical records. Most of the above items were investigated in the interview upon enrolment in the VEdeTTE1 cohort, thereby allowing a comparison to be made between the social and health status of the patients in the two time periods.

Information on treatment is collected from clinical records and recorded on a specific Registration Form beginning with the first treatment received during the VEdeTTE1 study period until the end of the VEdeTTE2 study. Details are collected on each treatment: type, dose, start and end dates. All types of treatment used in the NHS treatment centres are recorded: pharmacological treatments (methadone and buprenorphine maintenance or detoxification, psychotropic drugs, naltrexone, detoxification with non-opiate drugs), psychosocial treatments (counselling, social advice, psychotherapy, job guidance), in-patient detoxification, semi-residential treatment and residential therapeutic community.

### 2.5 Biological sample

Participating patients are also required to provide a sample of nape, axillary or pubic hair. Hair analysis is performed to ascertain the reliability of self-reported drug use [29-34]. In order to have information on drug use in the last 30 days, and as hair grows between 0.8 and 1.4 cm per month, interviewers collect hair from the nape if at least one cm long, otherwise from the pubic (preferably) or axillary areas. Morphine and 6-monoacetylmorphine are examined as markers of heroin use; cocaine and its metabolite benzoylecgonine are the markers of cocaine use [35,36]. The test results are qualitative (positive/negative), with a quantitative measure (ng/mg of hair) for specimens that screened positive or uncertain. To give a reliable estimate of the last 30 days use, both the length of the sample and the site of the body from which hair is collected will be taken into account at the statistical analysis stage.

## 3 Pilot study

A pilot study was carried out to verify the feasibility of the follow-up procedures. The specific objectives of the pilot study were:

- to assess the feasibility of the VEdeTTE2 study with regard to the effectiveness of contact procedures and patients' acceptance rate;

- to compare the contact rate of external interviewers and NHS drug abuse treatment centre personnel;

- to test the acceptability of study instruments (questionnaire, biological sample, monitoring form), for both patients and interviewers;

- to evaluate the reliability of self-reported drug use, by comparison with hair test results.

The pilot study was carried out in the Piedmont region between April and June 2001, involving 10 NHS drug abuse treatment centres. Piedmont was chosen based on the availability of a regional ad hoc fund; moreover, 26% of the participants in the VEdeTTE1 study were in Piedmont, and this subgroup was representative of the entire cohort. From these 1,062 patients, 60 were selected for the pilot study: all new patients and a random sample of re-entry and prevalent patients. To assess the difference in contact rate, patients were randomly assigned to three external interviewers (29 patients) and three NHS treatment centre personnel (31 patients).

### 3.1 Results

At the beginning of the pilot phase, an average of 28 months after enrolment in VEdeTTE1, 51 (85%) patients were still attending NHS treatment centres, of whom 40 (78.4%) were interviewed, eight (15.7%) refused and three (5.9%) were not traced. Of the nine patients no longer attending treatment centres, only one (11.1%) was interviewed, two (22.2%) refused, one (11.1%) was dead, one (11.1%) was living abroad and four (44.4%) were not traced. The proportion not traced among patients no longer attending NHS treatment centres (44.4%) was significantly higher than among those still in attendance (5.9%) (Fisher's exact test p = 0.007).

Five of the six patients living in a therapeutic community were interviewed. The only patient in prison was declared a drop-out, as it was impossible to contact the patient, although many attempts had been made. The two patients who had migrated to another Italian region were searched for by telephone and through their acquaintances; one of them was contacted but not interviewed, because of administrative problems.

By the end of the pilot study, 53 patients (88.3%) had been traced; 41 (68.3%) had been interviewed, 10 (16.6%) had refused, one (1.7%) was dead, one was living abroad (1.7%), and seven (11.7%) were untraceable (Table 1). A description of the 41 patients who were interviewed for the pilot study is given in table 2. The median follow-up period at the time of the interview was 29 months.

**Table 1:** Pilot study tracing results, by sex and patient classification

	**Consent**	**Refusal**	**Dead**	**Abroad**	**Total traced**	**Dropped-out**	**Total**
	**n (%)**	**n (%)**	**n (%)**	**n (%)**	**n (%)**	**n (%)**	**n (%)**

Males	25 (67.6)	7 (18.9)	0 (0.0)	1 (2.7)	33 (89.2)	4 (10.8)	37 (100)
Females	16 (69.6)	3 (13.0)	1 (4.3)	0 (0.0)	20 (87.0)	3 (13.0)	23 (100)
New	16 (66.7)	3 (12.5)	0 (0.0)	1 (4.2)	20 (83.4)	4 (16.7)	24 (100)
Re-entry	2 (100)	0 (0.0)	0 (0.0)	0 (0.0)	2 (100)	0 (0.0)	2 (100)
Prevalent	23 (67.6)	7 (20.6)	1 (2.9)	0 (0.0)	31 (91.2)	3 (8.8)	34 (100)
Total	41 (68.3)	10 (16.6)	1 (1.7)	1 (1.7)	53 (88.3)	7 (11.7)	60 (100)

**Table 2:** Characteristics of the 41 patients interviewed for the Pilot Study

**Characteristic**	**N**	%*
**Gender**		

Males	25	61.0
Females	16	39.0

**Typology of patients**		

New	11	26.8
New for VEdeTTE1 study	5	12.2
Re-entry	2	4.9
Prevalent	23	56.1

**Education**		

< 9 years of school	29	70.7
9 – 13 years of school	11	26.8
> 13 years of school	0	0.0
Missing	1	2.4

**Employment**		

Steady job	24	58.5
Temporary job	3	7.3
Unemployed/non-working status	12	29.3
Missing	2	4.9

Self-reported use in the last 30 days**		

Heroin	19	46.3
Cocaine	11	26.8
Ecstasy	1	2.4
Cannabis	8	19.5
Alcohol	10	24.4

**Age**	**Mean**	**SD**

Age at time of VEdeTTE2 interview	32.9	6.2
Age at first heroin use	19.3	4.7
Age at first NHS drug abuse treatment	24.6	5.8

* proportion out of the total number of interviewed patients (n = 41)

** multiple answers

The difference in contact rate between external interviewers (100%, 29/29) and NHS treatment centre personnel (71%, 22/31) was statistically significant (Fisher's exact test p = 0.002), even after excluding the two untraceable patients (one dead and one living abroad) who had been both assigned to NHS treatment centre personnel. There was no difference, however, between the proportions of patients interviewed among those that had been traced: 79% (23/29) and 82% (18/22) respectively.

All except one of the 41 patients interviewed provided a biological sample (33 nape hair, 5 axillary hair, 2 pubic hair). Two samples (5%, 1 nape and 1 axillary hair) were not analysed because the quantity was inadequate; seven samples (17.5%, 4 nape and 3 axillary hair) screened negative owing to an inadequate quantity or inhomogeneous length. The axillary hair samples were not reliable: most of them were inadequate in quantity or inhomogeneous in length. Of the remaining 31 samples, 58.1% screened positive for heroin (18/31) and 67.7% for cocaine (21/31). For heroin, sensitivity was 78% and specificity 85%; for cocaine, sensitivity and specificity were 43% and 100% respectively (Table 3).

**Table 3:** Heroin and cocaine self-reported use compared to hair sample test results

	Biological sample			
	
	Heroin		Cocaine	
Self-reported drug use in the last 30 days	Positive	Negative	Positive	Negative

yes	14	2	9	0
no	4	11	12	10

Total agreement	80.7%		61.3%	
Sensitivity	77.8%		42.9%	
Specificity	84.6%		100%	

### 3.2 Improvements to procedures in the VEdeTTE2 study

On the basis of the pilot phase, the VEdeTTE2 study was judged feasible. Contact procedures succeeded in tracing 88% (53/60) of patients, consistent with other studies [37], and the acceptance rate was good (77%, 41/53). For the biological sample, the acceptance rate was unexpectedly high (97.6%, 40/41).

The interviewer incentive scheme was successful. However, to encourage interviewers to obtain the consent of as many patients as possible and to avoid the temptation to contact only those patients still attending the NHS treatment centres, the interviewer reward was changed slightly in the main study. Interviewers received 90 euros for every completed interview, with an extra 100 euros for each additional interview after 50% of their assigned patients had been interviewed; and 5 euros for each refusal or drop-out.

As treatment centre personnel had a lower contact rate than external interviewers, it was decided that no more than half of the interviewers would be NHS treatment centre personnel, and that they would be assigned fewer patients compared to external interviewers. To overcome the difficulties encountered in contacting the patient in prison, a request for official authorization was sent to the Ministry of Justice. The Ministry of Justice sent a statement of collaboration to all the prisons involved to facilitate the work of the interviewers.

Other changes were introduced: the monitoring forms were simplified accordingly to the comments provided by the interviewers; the sections in the questionnaire were re-ordered with questions about demographic characteristics at the beginning, and the more intrusive ones (e.g. drug use, overdose, health status) toward the end; instructions for both the questionnaire and the collection of the biological sample were improved. Moreover, in order to deepen the investigation into illegal activity, a twelfth section was developed that included questions about criminal activity and prostitution. This section was on a separate sheet, which was given to the patient to be completed and returned by regular mail. The hope was that this procedure would encourage patients to answer honestly, without fear of legal consequences.

Sensitivity of the biological test was quite good for heroin (78%), but not for cocaine (42.3%). This may be owing to the inconsistency between the length of the hair examined (2–4 cm corresponding to 2–4 months) and the recall period in the questionnaire (30 days). Patients who had used drugs two or three months prior to the interview but not within the last 30 days would have screened positive and given a negative response on the questionnaire. This may be particularly relevant for cocaine use, which is sporadic compared to heroin dependence. On the other hand, the fact that two patients reported heroin use on the questionnaire but screened negative may suggest that the biological test lacks the power to detect sporadic drug use. Moreover, even though all samples that screened positive were confirmed by gas chromatography-mass spectrometry, certain laboratory errors, such as false positives caused by contamination in the laboratory, could have been more thoroughly addressed had there been more specimens. This is unlikely to affect the final results in the VEdeTTE2 main study, as there were a large number of samples. In order to improve the results in the main study, only the two centimetres of hair nearest the root was tested and patients were asked about their drug use in the last two months.

## 4 Discussion

In Italy, there have not been any large follow-up studies of heroin addicts evaluating rehabilitation outcomes in the long term. The scarcity of international data on long-term rehabilitation outcomes and the availability of a large sample such as the VEdeTTE1 cohort as a source of potential subjects were the reasons for undertaking the follow-up study. Moreover, the VEdeTTE1 study, because of its study design, could investigate the effect of different treatments and combinations of treatments on retention and mortality [38], but not on drug use. The VEdeTTE2 follow-up study, which combines self-reported measures with the results of a biological sample, will provide a more reliable assessment of drug use and, therefore, is a good occasion for a thorough investigation into the effects of treatments on long-term drug use.

The aim of this paper is to describe the VEdeTTE2 study design and protocol, and to present the results of the pilot study underlining the changes to the instruments consequently implemented.

The results of the pilot phase pointed out the validity of the procedures designed to limit attrition: the number of traced subjects was satisfactory (88%). Moreover, the pilot phase was very useful in identifying possible causes of delays and attrition. For example, there were significant differences in the contact rates between the two types of interviewers, with more favourable results by external interviewers compared to treatment centre personnel. This could be probably due to differences in the availability of dedicated time between the two categories of interviewers. In consequence of this result, the number of NHS interviewers was limited to 50% of the total.

Study instruments contained some flaws, identified by the interviewers, which were consequently modified.

The self-reported drug use is a crucial item of a study aimed at measuring the use of substances. For this reason, it is generally recommended to collect a biological sample in order to have a reliable measure of drug use. In the pilot phase, sensitivity of the biological test was quite good for heroin (78%) but lower for cocaine (42.3%). This result confirmed the need to obtain a hair sample from all patients.

These results underline the importance of the pilot phase when planning a follow-up study, especially when heroin addicted are involved.

## Competing interests

The author(s) declare that they have no competing interests.

## Authors' contributions

FV-T and FM drafted the article and performed the statistical analysis in the pilot study. RD, PB, EV, MD, FF conceptualized the VEdeTTE2 study; AI, FL and FM were in charge of the pilot study and were responsible for the main study field work; PZ and PR organized and performed the analysis of the biological samples. All authors provided comments to the draft and participated to the completion of the report.
